# Catheter ablation for AF improves global thrombotic profile and enhances fibrinolysis

**DOI:** 10.1007/s11239-017-1548-3

**Published:** 2017-09-18

**Authors:** Maria Niespialowska-Steuden, Vias Markides, Mohamed Farag, David Jones, Wajid Hussain, Tom Wong, Diana A. Gorog

**Affiliations:** 1 National Heart & Lung Institute, Imperial College, Dovehouse Street, London, SW3 6LY UK; 2 Postgraduate Medical School, University of Hertfordshire, Hatfield, UK; 3 Department of Cardiology, East and North Hertfordshire NHS Trust, Hertfordshire, UK; 4 Royal Brompton & Harefield NHS Foundation Trust, London, UK

**Keywords:** Atrial fibrillation, Thrombosis, Fibrinolysis, Ablation, Cardioversion

## Abstract

Patients with atrial fibrillation (AF) are at increased risk of thrombotic events despite oral anticoagulation (OAC). Radiofrequency catheter ablation (RFCA) can restore and maintain sinus rhythm (SR) in patients with AF. To assess whether RFCA improves thrombotic status. 80 patients (71% male, 64 ± 12y) with recently diagnosed AF, on OAC and scheduled to undergo RFCA or DC cardioversion (DCCV) were recruited. Thrombotic status was assessed using the point-of-care global thrombosis test (GTT), before, and 4–6 weeks after DCCV and 3 months after RFCA. The GTT first measures the time taken for occlusive thrombus formation (occlusion time, OT), while the second phase of the test measures the time taken to spontaneously dissolve this clot through endogenous thrombolysis (lysis time, LT). 3 months after RFCA, there was a significant reduction in LT (1994s [1560; 2475] vs. 1477s [1015; 1878]) in those who maintained SR, but not in those who reverted to AF. At follow-up, LT was longer in those in AF compared to those in SR (AF 2966s [2038; 3879] vs. SR 1477s [1015; 1878]). RFCA resulted in no change in OT value, irrespective of rhythm outcome. Similarly, there was no change in OT or LT in response to DCCV, irrespective of whether SR was restored. Successful restoration and maintenance of SR following RFCA of AF is associated with improved global thrombotic status with enhanced fibrinolysis. Larger studies are required to confirm these early results and investigate whether improved thrombotic status translates into fewer thromboembolic events.

**Electronic supplementary material:**

The online version of this article (doi:10.1007/s11239-017-1548-3) contains supplementary material, which is available to authorized users.

## Introduction

Patients with atrial fibrillation (AF) exhibit a prothrombotic state and are at increased risk of thromboembolic events, which is reduced but not abolished by oral anticoagulation (OAC) [1, 2]. Left atrial thrombus formation in patients with AF is thought to be determined by the components of Virchow’s triad, namely blood stasis (due to loss of atrial contractility and dilatation of the left atrium), endothelial injury (through myocyte hypertrophy, sclerosis and fibroelastosis) and a prothrombotic/hypercoagulable state [2, 3]. Endogenous fibrinolysis is a natural defense mechanism against lasting thrombus formation, such that the occurrence of thrombus is determined by the balance between procoagulant factors on the one hand, and effectiveness of fibrinolysis on the other. Radiofrequency catheter ablation (RFCA) can restore and maintain sinus rhythm (SR) long-term in patients with AF, with far superior efficacy compared to DCCV. Given that blood stasis due to reduced atrial contractility is an important component of Virchow’s triad, it could be postulated that restoration of atrial contraction in AF patients through restoration of SR with RFCA may favourably reduce the prothrombotic state. It is acknowledged that additional factors including systemic inflammation may also contribute to a procoagulant state, which is one of the reasons for the current recommendation for continued anticoagulant therapy after successful RFCA. Superiority of RFCA, compared to a rate-control strategy, in improving symptoms and quality of life as well as the haemodynamic benefit in patients with heart failure, is well documented [4, 5]. However, whether maintenance of SR in AF patients can reduce thrombotic risk, is incompletely understood. Large studies, most notably the AFFIRM (atrial fibrillation follow-up investigation of rhythm management) trial, have not shown a mortality or stroke reduction benefit with rhythm control (achieved with antiarrhythmic medications and cardioversion) compared to rate control [6]. However, many more patients in the rhythm control arm had discontinued anticoagulation and a post-hoc analysis indicated a trend for mortality benefit following restoration of SR [7]. Non-randomized data indicate normalization of stroke risk following successful AF ablation, suggesting that treating the substrate of AF may favourably improve thrombotic profile [8]. The prothrombotic/hypercoagulable state in AF comprises enhanced platelet activation, evidenced by raised levels of soluble P-selectin, β-thromboglobulin and platelet factor four released from platelet alpha granules; as well as raised levels of von Willebrand factor released by the endothelium, which mediate subendothelial platelet adhesion; enhanced coagulation, through increased levels of prothrombin fragments 1 and 2 and thrombin-antithrombin III complex; and impaired fibrinolysis [3, 9]. The fibrinolytic system plays an important role in regulating local thrombus formation. Increased level of tissue-type plasminogen activator antigen and tissue-type plasminogen activator inhibitor-1 has been documented in AF [9] which is reduced significantly with anticoagulation [9]. D-dimer levels, reflecting fibrin turnover, are elevated in patients with AF, especially those with left atrial appendage thrombi and are reduced by anticoagulation [10]. We aimed to assess whether restoration of SR in patients with AF may have favourable effects on thrombotic profile, and whether modification of the substrate of AF, through ablation improves thrombotic status more than simple restoration of SR through cardioversion. We used a method to assess thrombotic status that would capture changes in platelet activation, coagulation, and fibrinolysis, which are felt to belie the prothrombotic/hypercoagulable state in AF. To further assess whether changes in thrombotic status were systemic or local to the left atrium, we compared blood from the right and left atria with peripheral blood, in patients with and without AF undergoing ablation.

## Methods

### Patients, clinical procedures and follow-up

The study was approved by the National Research Ethics Service. 80 patients with AF scheduled to undergo direct current cardioversion (DCCV) (n = 40) or RFCA (n = 40) were enrolled after obtaining written informed consent. The exclusion criteria in Table 1 were applied. Venous blood was taken at 2 time-points from each patient; at baseline and at post-procedural follow up. Patients acted as self-controls.

**Table 1:** Exclusion criteria

Exclusion criteria
Age <18 years
Impaired renal function with eGFR < 30 mL/min (since renal failure is associated with platelet function defect that may confound results)
Persons who, in the opinion of the investigator, have significant neurological, hepatic, renal, endocrine, cardiovascular, gastrointestinal, pulmonary, haemorrhagic, metabolic or other disease likely to confound the study results
Persons with a history of substance abuse or who demonstrates signs or clinical features of active substance abuse or psychiatric disease
Alcohol consumption above recommended safe levels (i.e. above 21 units per week for males, or 14 units per week for females) due to the potential effects of high alcohol levels on platelet reactivity
Any illness deemed significant by the investigator during the 4 weeks preceding the screening period of the study
Blood dyscrasia (platelets <70 × 10^9^/l, Hb <80 g/dl, leucocyte count <3.5 × 10^9^/l, neutrophil count <1 × 10^9^/l), or known bleeding disorder
Subject currently enrolled in an investigational device or drug trial

*eGFR* estimated glomerular filtration rate, *Hb* haemoglobin, *UNL* upper normal limit

Baseline demographics and clinical characteristics were obtained from patients, review of case notes and electronic patient records, at baseline and at follow-up, which was approximately 4–6 weeks after DCCV and 3 months after RFCA. The end-study visit involved assessment of thrombotic status, review of the 24-h Holter monitor and ECG results, and verification of other clinical characteristics.

Patients had been therapeutically anticoagulated for at least 4 weeks pre-DCCV. Non-vitamin K oral anticoagulants (NOAC) were taken on the day of the procedure. Cardioversion was performed according to standard local protocol under general anesthesia, delivering up to three biphasic synchronized shocks. 29 patients were successfully cardioverted to SR. Digoxin was stopped if SR was restored, but other medications were continued. At 6 weeks’ follow-up, 9 patients had AF recurrence, as evidenced by 24-h Holter monitoring and ECG. In patients taking warfarin, INR levels were similar before DCCV and at follow-up (2.5 ± 0.33 vs. 2.4 ± 0.40; p = 0.24).

Patients undergoing RFCA had been therapeutically anticoagulated for at least 4 weeks. The morning dose of dabigatran was taken the morning of the day before the procedure. RFCA was performed according to standard local protocol under general anesthesia. All procedures were performed with a 3D mapping system, either CARTO3 (Biosense, Diamond Bar, CA, USA) or Velocity (St Jude Medical), alongside conventional mapping systems and irrigated ablation catheters. Wide area circumferential ablation with pulmonary vein isolation was performed in all patients, accompanied by linear ablation in the left and/or right atria with validation of line integrity, and limited electrogram-based ablation as clinically indicated. There were no major peri-procedural complications. No changes to medications were made post-procedure. At 3 months, AF recurred in 10 out of 40 RFCA patients. INR levels at baseline and follow-up were similar (2.37 ± 0.44 vs. 2.33 ± 0.40; p = 0.67).

### Blood sampling

Peripheral blood samples were taken on 2 occasions: (1) just before DCCV or RFCA, and (2) at follow-up, approximately 4–6 weeks after DCCV and 3 months after RFCA. Blood was obtained from an ante-cubital vein using an 18-G butterfly cannula with a two-syringe technique, taking care to avoid prolonged tourniquet time. The two-syringe technique involved discarding the first 5 mL of blood and using the second 5 mL for assessment of thrombotic status.

### Assessment of thrombotic status

Assessment of thrombotic status was performed using the global thrombosis test (GTT) (Thromboquest Ltd., London, UK). This automated point-of-care test sequentially assesses both platelet reactivity and endogenous thrombolysis from a native blood sample. The patient was positioned in close proximity to the GTT such that the blood sample was injected into the instrument within 15 s of withdrawal and the automated measurement begun. The GTT assesses the time taken to form an occlusive platelet thrombus (occlusion time, OT), and in the second phase of the test, measures the time required to dissolve the thrombus formed in the first phase, through endogenous thrombolysis, which restores flow (lysis time, LT). The principle of the GTT has previously been described [11]. In brief, blood flows under physiological conditions through narrow gaps where it is subjected to high shear, and the instrument measures the time (d) between two consecutive blood drops downstream of this. As shear-induced platelet aggregation causes thrombus to form near the gaps, the time interval gradually increases as flow slows down and at an arbitrary point (d ≥ 15 s, before reaching complete occlusion), the end point of the measurement is displayed. The maximum time allowed for occlusive thrombus formation was pre-set to 800 s. Following this, there is a preset “thrombus stabilization time” of 200 s, during which the sensors are inactive. This time allows stabilization of the formed thrombi to allow lasting occlusion and ignores small re-bleeds. Restart of blood flow after occlusion is due to spontaneous thrombolysis. If lysis does not occur until >6000 s (LT cut-off time), “no lysis” is recorded. The coefficient of variation (cv) was assessed by testing a cohort of 10 stable patients on two occasions, 48 h apart.

### Chamber-specific assessment of thrombotic status

In order to assess whether the prothrombotic state that exists in the left atrium of patients with AF is a local or systemic phenomenon, and to assess the chamber-specific differences in thrombotic status, in a separate group of 20 patients undergoing RFCA of AF or left atrial tachycardia for conventional clinical indications, thrombotic status was assessed in blood taken peripherally and from intra-cardiac chambers (Supplementary Table 1) at baseline before ablation. Blood samples were obtained from the femoral vein, right atrium and left atrium (prior to heparin administration) at the beginning of the procedure. Ablation was performed as above, according to standard protocols. Additional sampling was performed after heparin administration and at the end of the procedure (after administration of protamine to reverse heparin). Further samples were taken 4 h after ablation and at 3 months’ follow-up from a peripheral vein.

### Statistical analysis

Since this was a pilot study and patients acted as self-controls with pre- and post- intervention sampling to minimise any bias or confounders, samples of 40 patients per group were felt to be sufficient. Data are presented as mean and standard deviation for normally distributed or median [interquartile range] for skewed continuous variables and as proportions for categorical variables. Differences in OT/LT before and after DCCV/RFCA within one group were analyzed by paired t-test or Wilcoxon’s method. Between group comparisons for continuous variables were assessed using a t-test or Mann–Whitney U test. The Kruskal–Wallis rank test was used to compare differences in baseline thrombotic status between patients with different types of AF. Dichotomous variables were compared using Chi square test with continuity correction or Fisher’s exact test, as appropriate. Correlations were performed using Spearman’s rank correlation. Univariate and multivariate linear regression models were used to identify the independent predictors of impaired fibrinolysis in the DCCV and ablation cohorts. All the study variables listed in Tables 2 and 3 were first analysed with univariate analysis and those that showed a significant interaction (p < 0.05) in the univariate analysis were entered into the multivariate analysis. Statistical significance was fixed at 0.05. Analyses were performed with STATISTICA software (StatSoft, version 10).

**Table 2:** Baseline characteristics of patients undergoing direct current cardioversion (DCCV) and radiofrequency ablation (RFCA), n (%)

Patient characteristic	DCCV cohort (n = 40)	RFCA cohort (n = 40)	p value
Age, years	63.9 ± 11.6	64 ± 13	0.534
Male	29 (72)	28 (70)	0.851
BMI	28.7 ± 6	28.5 ± 5	0.793
Paroxysmal AF	N/A	10 (25)	N/A
Persistent AF	19 (48)	9 (23)	0.034
Long-standing persistent AF	21 (53)	21 (53)	1
Medical history			
Hypertension	22 (55)	11 (27.5)	0.023
Diabetes mellitus	5 (13)	5 (13)	0.996
Hyperlipidemia	18 (45)	17 (42)	0.851
Metabolic syndrome	16 (40)	10 (25)	0.188
Coronary artery disease	4 (10)	8 (20)	0.444
Prior stroke/TIA	2 (5)	4 (10)	0.703
Chronic kidney disease (eGFR < 60 mL/min/1.73 m^2^)	5 (13)	5 (13)	0.996
Echocardiographic characteristics			
Ejection fraction >55%	20 (50)	30 (75)	0.284
Ejection fraction 45–54%	18 (45)	7 (18)	0.067
Ejection fraction <44%	2 (5)	3 (8)	0.703
No atrial dilatation	11 (27)	6 (15)	0.703
Mild atrial dilatation (LA diameter 4.1–4.6 cm)	18 (45)	25 (63)	0.179
Moderate atrial dilatation (LA diameter 4.7–5.2 cm)	9 (23)	5 (13)	0.179
Severe atrial dilatation (LA diameter >5.3 cm)	2 (5)	4 (10)	0.703
Risk scores			
CHA_2_DS_2_VASc	1.9 ± 1.39	1.9 ± 1.7	0.798
HASBLED	1.25 ± 0.83	1.2 ± 0.7	0.754
Medications			
Warfarin	20 (50)	36 (90)	0.116
Apixaban	2 (5)	0 (0)	N/A
Rivaroxaban	1 (3)	0 (0)	N/A
Dabigatran	17 (42)	4 (10)	0.013
Beta-blocker	31 (77)	21 (53)	0.179
Calcium channel blocker	10 (25)	6 (15)	0.444
Digoxin	6 (15)	1 (2.5)	0.338
Amiodarone	1 (3)	6 (15)	0.338
Statin	13 (32)	17 (43)	0.444
ACE inhibitor	11 (27)	6 (15)	0.338
ARB	8 (20)	6 (15)	0.703
Laboratory characteristics			
Haemoglobin (g/L)	144.6 ± 15	139 ± 17	0.186
Haematocrit (%)	0.43 ± 0.04	0.41 ± 4	0.159
Platelet count (×10^9^/L)	234 ± 83	212 ± 58	0.508
INR (warfarin patients)	2.32 ± 0.47	2.3 ± 0.4	0.099
Creatinine (μmol/L)	83.2 ± 14.5	95 ± 84	0.411
eGFR (mL/min/1.73 m^2^)	87.6 ± 25	96.6 ± 28	0.145
Total cholesterol (mmol/L)	5.2 ± 1.2	5.0 ± 1.7	0.428
CRP (mg/L)	3.8 ± 4.53	3.52 ± 4.18	0.778
Fibrinogen (g/L)	4.1 ± 1.14	4.06 ± 1.64	0.893
Baseline OT (s)	488 [396–573]	524 [445–641]	0.123
Baseline LT (s)	1819 [1453–2180]	2015 [1507–2740]	0.148

Left ventricular ejection fraction was calculated by Simpson’s method

*ACE* angiotensin-converting enzyme, *ARB* angiotensin receptor blocker, *BMI* body mass index, *CRP* C-reactive protein, *eGFR* estimated glomerular rate, *INR* International Normalized Ratio, *LA* left atrium, *N*/*A* not applicable, *TIA* transient ischaemic attack

Normal values: haemoglobin 130–180 g/L in men and 115–165 g/L in women; haematocrit 40–52% in men and 36–47% in women; platelet count 150–400 × 10^9^/L; INR range 2.0–3.0; creatinine 60–110 μmol/L in men and 45–90 μmol/L in women; eGFR: above 90 mL/min/1.73 m^2^; total cholesterol <5.2 mmol/L; CRP 0–10 mg/L; fibrinogen 2–5 g/L

**Table 3:** Baseline clinical characteristics of direct current cardioversion (DCCV) cohort, with respect to rhythm outcome at follow-up

Patient characteristic	DCCV cohort (n = 40)	AF at follow-up (n = 20)	SR at follow-up (n = 20)	p value
Age, years	63.9 ± 11.6	62.5 ± 12	65.2 ± 11.5	0.472
Male	29 (72%)	15 (75)	14 (70)	0.731
BMI	28.7 ± 6	29.6 ± 7	27.5 ± 4.3	0.358
Medical history				
Hypertension	22 (55)	12 (60)	10 (50)	0.537
Diabetes mellitus	5 (13)	1 (5)	4 (20)	0.159
Hyperlipidemia	18 (45)	7 (35)	11 (55)	0.213
Metabolic syndrome	16 (40)	7 (35)	9 (45)	0.821
Coronary artery disease	4 (10)	2 (10)	2 (10)	1
Prior stroke/TIA	2 (5)	1 (5)	1 (5)	1
Chronic kidney disease (eGFR < 60 mL/min/1.73 m^2^)	5 (12.5)	1 (5)	4 (20)	0.159
Echocardiographic characteristics				
Left ventricular function				
Ejection fraction >55%	20 (50)	9 (45)	11 (55)	0.539
Ejection fraction 45–54%	18 (45)	9 (45)	9 (45)	1
Ejection fraction <44%	2 (5)	2 (10)	0 (0)	0.154
No atrial dilatation	11 (27)	4 (20)	7 (35)	0.3
Mild atrial dilatation (LA diameter 4.1–4.6 cm)	18 (45)	9 (45)	9 (45)	1
Moderate atrial dilatation (LA diameter 4.7–5.2 cm)	9 (22.5)	6 (30)	3 (15)	0.267
Severe atrial dilatation (LA diameter >5.3 cm)	2 (5)	1 (5)	1 (5)	1
Risk scores				
CHA_2_DS_2_VASc	1.9 ± 1.39	1.9 ± 1.2	1.9 ± 1.6	1
HASBLED	1.25 ± 0.83	1.3 ± 0.8	1.2 ± 0.89	0.711
Medications				
Warfarin	20 (50%)	9 (45)	11 (55)	0.539
Apixaban	2 (5)	0 (0)	2 (10)	N/A
Rivaroxaban	1 (2.5)	1 (5)	0	N/A
Dabigatran	17 (42)	11 (55)	6 (30)	0.115
Beta-blocker	31 (77)	15 (75)	16 (80)	0.713
Calcium channel blocker	10 (25)	7 (35)	3 (15)	0.151
Digoxin	6 (15)	4 (20)	2 (10)	0.388
Amiodarone	1 (2.5)	0 (0)	1 (5)	0.323
Statin	13 (32)	4 (20)	9 (45)	0.095
ACE inhibitor	11 (27)	8 (40)	3 (15)	0.08
ARB	8 (20)	5 (25)	3 (15)	0.692
Laboratory characteristics				
Haemoglobin (g/L)	144.6 ± 15	147 ± 14	141 ± 16.7	0.318
Haematocrit (%)	0.43 ± 0.04	0.43 ± 0.04	0.42 ± 0.05	0.692
Platelet count (×10^9^/L)	234 ± 83	251 ± 101	208 ± 35.6	0.192
INR (warfarin patients)	2.54 ± 0.33	2.59 ± 0.37	2.47 ± 0.28	0.673
Creatinine (μmol/L)	83.2 ± 14.5	85 ± 9.5	81.2 ± 18	0.382
eGFR (mL/min/1.73 m^2^)	87.6 ± 25	85.8 ± 21	89 ± 29	0.661
Total cholesterol (mmol/L)	5.2 ± 1.2	5.7 ± 1.7	4.8 ± 0.5	0.266
CRP (mg/L)	3.8 ± 4.53	4.25 ± 4.71	3.35 ± 4.42	0.537
Fibrinogen (g/L)	4.1 ± 1.14	3.93 ± 1.06	4.28 ± 1.21	0.34

Values are mean ± standard deviation or n (%). Left ventricular ejection fraction was calculated by Simpson’s method

*ACE* angiotensin-converting enzyme, *ARB* angiotensin receptor blocker, *BMI* body mass index, *CRP* C-reactive protein, *eGFR* estimated glomerular filtration rate, *INR* international normalized ratio, *LA* left atrium, *TIA* transient ischaemic attack

Normal values: haemoglobin 130–180 g/L in men and 115–165 g/L in women; haematocrit 40–52% in men and 36–47% in women; platelet count 150–400 × 10^9^/L; INR range 2.0–3.0; creatinine 60–110 μmol/L in men and 45–90 μmol/L in women; eGFR: above 90 mL/min/1.73 m^2^; total cholesterol < 5.2 mmol/L; CRP 0–10 mg/L; fibrinogen 2–5 g/L

## Results

Clinical characteristics of the DCCV and RFCA cohorts are shown in Tables 2, 3 and 4. The cv for OT and LT was 8 and 10%, respectively. The variables in Tables 2, 3 and 4 were interrogated for relation to pre- and post-procedural OT and LT in each cohort. In the DCCV cohort, there was a trend for an inverse relationship between baseline OT and haemoglobin level (r = −0.495; p = 0.08) but not to other variables. In the RFCA cohort, there was a weak inverse correlation between baseline OT and BMI (r = −0.358; p = 0.025). Baseline LT correlated with creatinine (r = 0.331; p = 0.044). LT after RFCA correlated with creatinine (r = 0.504; p = 0.001) and weakly with haematocrit (r = 0.35; p = 0.049). Applying the Kruskal–Wallis rank test to the group as a whole (DCCV and RFA groups combined), we found that baseline OT was not different between patients with persistent AF, paroxysmal AF, and long-standing persistent AF (497 [402–571] vs. 640 [505–729] vs. 497 [420–603] respectively; p = 0.100). Similarly, baseline LT did not differ between patients with different types of AF (1769 [1527–2217] vs. 2128 [1706–4537] vs. 1852 [1346–2228] respectively; p = 0.275).

**Table 4:** Baseline clinical characteristics of radiofrequency catheter ablation (RFCA) cohort with respect to rhythm at follow-up

Patient characteristic	RFCA cohort (n = 40)	AF at follow-up n = 10	SR at follow-up n = 30	p value
Age, years	64 ± 13	67 ± 13	63.8 ± 14	0.418
Male	28 (70)	7 (70)	21 (70)	1.000
BMI	28.5 ± 5	31.6 ± 3.4	27.6 ± 5	0.064
Paroxysmal AF	10 (25)	3 (30)	7 (23)	0.766
Persistent AF	9 (23)	3 (30)	6 (20)	0.416
Long-standing persistent AF	21 (53)	4 (40)	17 (57)	0.444
Medical history				
Duration of AF > 1 year	29 (72.5)	7 (70)	22 (73)	1.000
Previous ablations	13 (32.5)	5 (50)	8 (26.7)	0.479
Hypertension	11 (27.5)	4 (40)	7 (23)	0.467
Diabetes mellitus	5 (12.5)	0	5 (16.7)	0.571
Hyperlipidemia	17 (42)	5 (50)	12 (40)	0.751
Metabolic syndrome	10 (25)	4 (40)	6 (18)	0.014
Coronary artery disease	8 (20)	2 (20)	6 (20)	1.000
Prior stroke/TIA	4 (10)	0	4 (13.3)	0.559
Chronic kidney disease (eGFR < 60 mL/min/1.73 m^2^)	5 (12.5)	2 (20)	3 (10)	0.598
Echocardiographic characteristics				
Ejection fraction >55%	30 (75)	8 (80)	22 (73.3)	1.000
Ejection fraction 45–54%	7 (17.5)	1 (10)	5 (16.7)	1.000
Ejection fraction <44%	3 (7.5)	1 (10)	3 (10)	1.000
No atrial dilatation	6 (15)	0	9 (30)	0.173
Mild atrial dilatation (LA diameter 4.1–4.6 cm)	25 (62.5)	7 (70)	18 (60)	0.781
Moderate atrial dilatation (LA diameter 4.7–5.2 cm)	5 (12.5)	2 (20)	0	0.077
Severe atrial dilatation (LA diameter >5.3 cm)	4 (10)	1 (10)	3 (10)	1.000
Risk scores				
CHA_2_DS_2_-VASc	1.9 ± 1.7	2.2 ± 1.6	1.83 ± 1.8	0.571
HASBLED	1.2 ± 0.7	1.1 ± 0.6	1.2 ± 0.7	0.691
Medications				
Warfarin	36 (90)	9 (90)	27 (90)	1.000
Dabigatran	4 (10)	1 (10)	3 (10)	1.000
Beta-blocker	21 (53)	2 (20)	19 (63)	0.189
Calcium antagonist	6 (15)	4 (40)	2 (7)	0.060
Digoxin	1 (3)	1 (10)	0	0.268
Statin	17 (43)	6 (60)	11 (37)	0.523
ACE inhibitor	6 (15)	3 (30)	3 (10)	1.000
ARB	6 (15)	1 (10)	5 (17)	1.000
Laboratory characteristics				
Haemoglobin (g/L)	139 ± 17	144 ± 16	137 ± 15	0.217
Haematocrit (%)	0.41 ± 4	0.42 ± 6	0.41 ± 4	0.326
Platelet count (×10^9^/L)	212 ± 58	227 ± 52	207 ± 59	0.244
INR (warfarin patients)	2.3 ± 0.4	2.3 ± 0.4	2.3 ± 0.4	0.967
Creatinine (μmol/L)	95 ± 84	93 ± 28	96 ± 10	0.137
eGFR (mL/min/1.73 m^2^)	96.6 ± 28	85 ± 21	100.8 ± 30	0.35
Total cholesterol (mmol/L)	5.0 ± 1.7	4.5 ± 0.9	5.2 ± 1.9	0.523
CRP (mg/L)	3.52 ± 4.18	3.3 ± 3.97	3.6 ± 4.31	0.847
Fibrinogen (g/L)	4.06 ± 1.64	4.35 ± 1.32	3.96 ± 1.74	0.53
Procedure characteristics				
Procedure duration (min)	163 ± 76	247 ± 64	140 ± 62	0.001
Ablation time (ms)	1856 ± 1408	3358 ± 1451	1446 ± 1108	0.002
Number of energy application	33 ± 24	56 ± 32	26 ± 17	0.005

Values are mean ± standard deviation or n (%). Left ventricular ejection fraction was calculated by Simpson’s method

*ACE* angiotensin-converting enzyme, *ARB* angiotensin receptor blocker, *BMI* body mass index, *CRP* C-reactive protein, *eGFR* estimated glomerular filtration rate, *INR* international normalized ratio, *LA* left atrium, *TIA* transient ischaemic attack

Normal values: haemoglobin 130–180 g/L in men and 115–165 g/L in women; haematocrit 40–52% in men and 36–47% in women; platelet count 150–400 × 10^9^/L; INR range 2.0–3.0; creatinine 60–110 μmol/L in men and 45–90 μmol/L in women; eGFR: above 90 mL/min/1.73 m^2^; total cholesterol <5.2 mmol/L; CRP 0–10 mg/L; fibrinogen 2–5 g/L

### Effect of AF ablation on thrombotic status

In the ablation cohort, 10 of the 40 patients had AF recurrence at follow-up, which was associated with longer ablation procedure duration (p = 0.001), longer ablation time (p = 0.002), and higher energy application (p = 0.005) (Table 4).

**Fig. 1:**
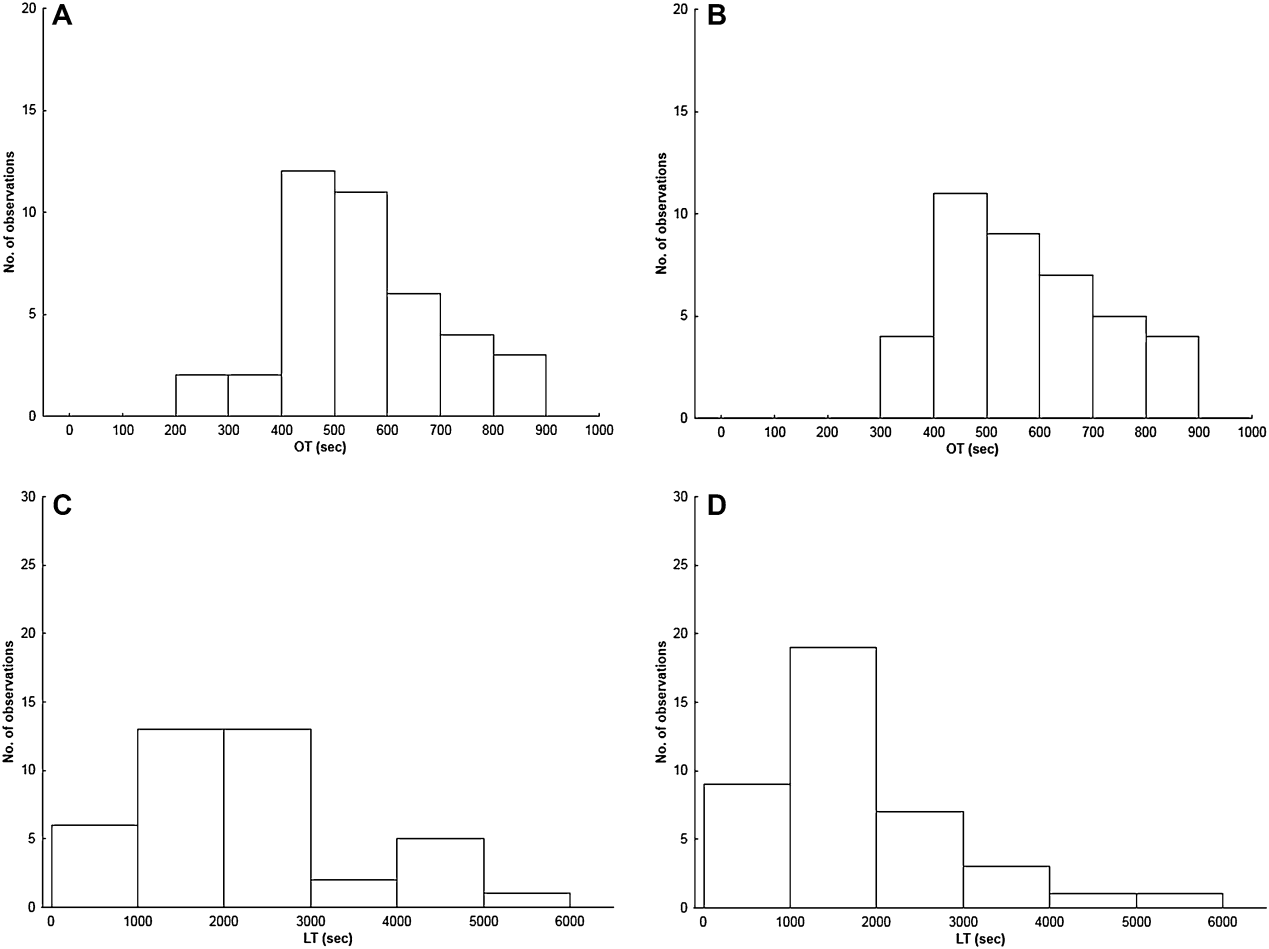
Distribution of occlusion time (OT) before (**a**) and after radiofrequency catheter ablation (RFCA) (**b**); and distribution of lysis time (LT) before (**c**) and after RFCA (**d**). *Y-axis* shows number of observations (subjects)

**Fig. 2:**
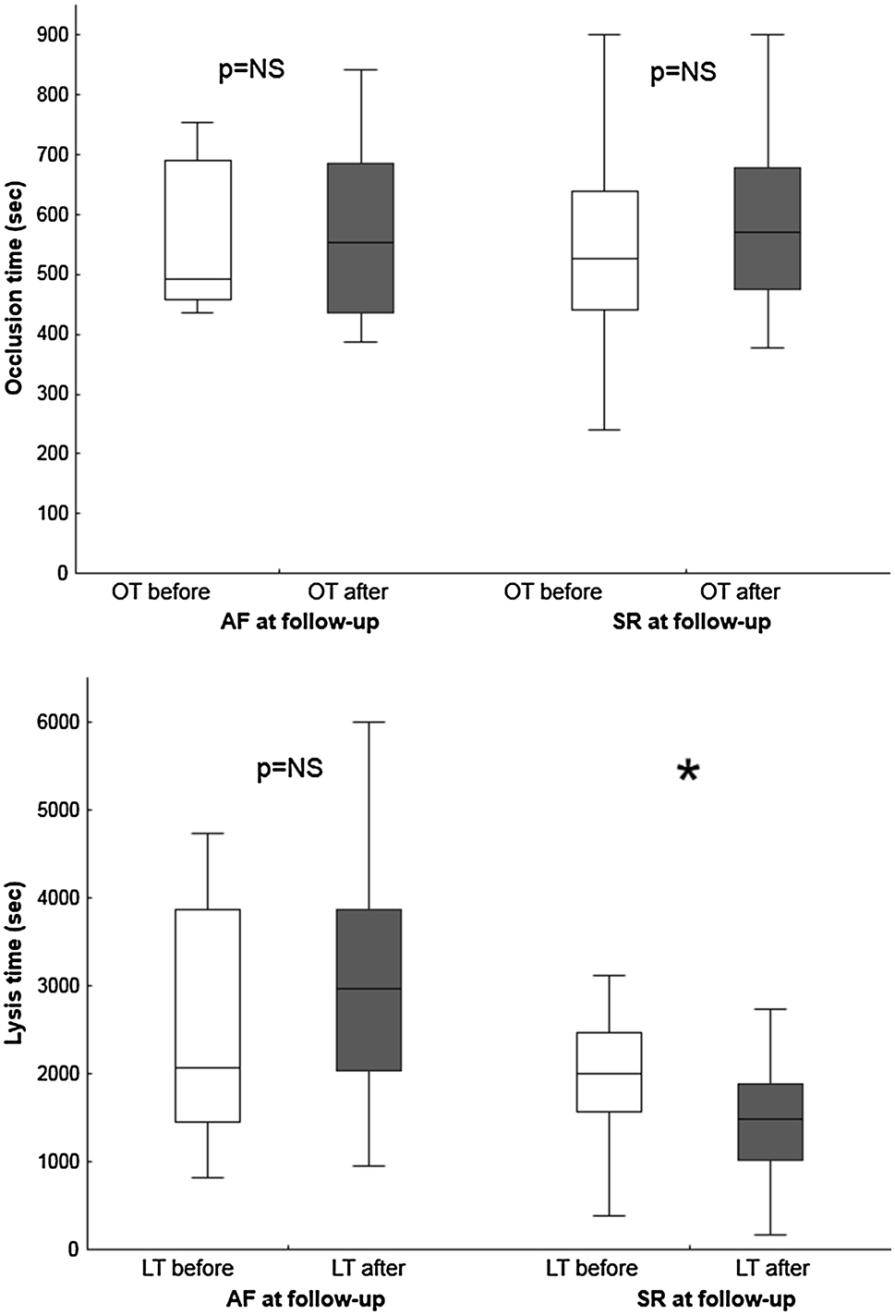
Occlusion time (OT) and Lysis time (LT) before and after AF ablation, with respect to rhythm at follow-up. Sinus rhythm (SR) *p < 0.001

In the group as a whole, there was no difference in OT before and after RFCA (523s [443; 640] vs. 571s [463; 682]; p = 0.139), regardless of whether patients were in AF at follow-up (493s [457; 691] vs. 552s [436; 685]; p = 0.514) or in SR (527s [440; 640] vs. 571s [476; 679]; p = 0.135) (Figs. 1, 2). At follow-up, there was no difference in OT between patients in SR and those in AF (571 s [476; 679] vs. 552 s [436; 685]; p = 0.9).

**Fig. 3:**
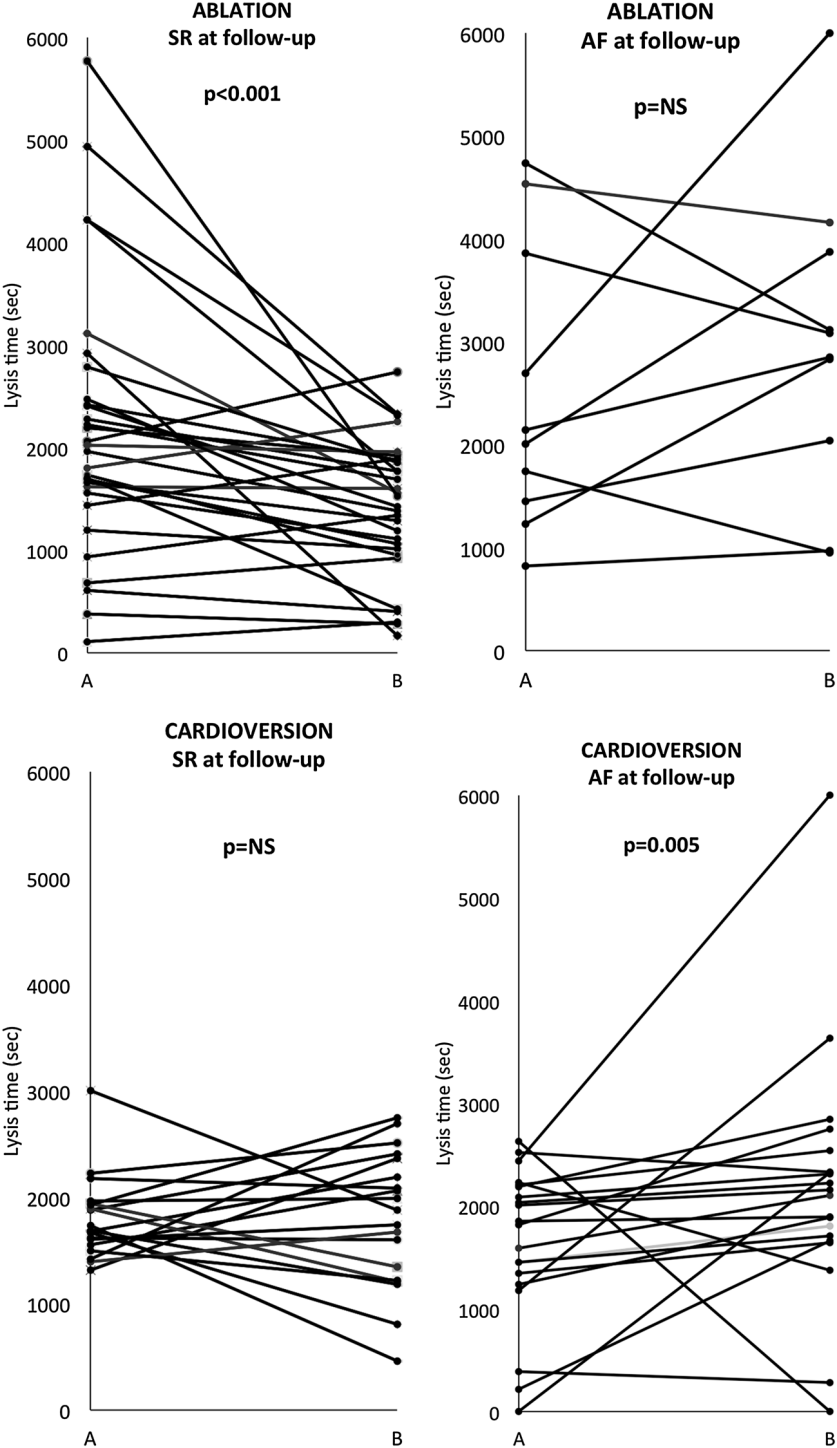
*Upper panel* change in lysis time before (**a**) and after (**b**) RFCA in individual patients, according to rhythm at follow-up. *Lower panel* change in lysis time before (**a**) and after (**b**) cardioversion in individual patients, according to rhythm at follow-up. Sinus rhythm (SR). *p < 0.001

In the RFCA cohort as a whole, LT was shorter after RFCA (2015s [1506; 2739] vs. 1645s [1037; 2285]; p = 0.014) (Figs. 1, 2). Among patients in SR at follow-up, there was a significant reduction in LT following RFCA (1994s [1560; 2475] vs. 1477s [1015; 1878]; p < 0.001), whereas there was no change in LT in those in AF at follow-up (2074s [1453; 3859] vs. 2966s [2038; 3879]; p = 0.500) (Figs. 2, 3). At follow-up, LT was significantly longer in those in AF compared to those in SR (2966s [2038; 3879] vs. 1477s [1015; 1878]; p = 0.002). Pre-ablation samples showed no baseline differences in LT between those who subsequently reverted to AF and those who maintained SR (2074s [1453; 3859] vs. 1994s [1560; 2475]; p = 0.606). Amongst patients undergoing RFCA, there was no difference between patients pre-and post-RFCA with respect to C-reactive protein (CRP, 3.52 ± 4.18 vs. 2.62 ± 3.01, p = 0.279) or fibrinogen (4.06 ± 1.64 vs. 4.39 ± 1.46, p = 0.337). In the subgroup who reverted to AF, there was no difference in CRP (3.3 ± 3.97 vs. 3.2 ± 3.15, p = 0.955) or fibrinogen (4.35 ± 1.32 vs. 4.48 ± 1.55, p = 0.847) before and after the procedure. In the subgroup who maintained SR, there was no difference in CRP (3.6 ± 4.31 vs. 2.43 ± 2.99; p = 0.223) or fibrinogen (3.96 ± 1.74 vs. 4.36 ± 1.46; p = 0.332) before and after the procedure.

In the ablation group, using univariate linear regression analysis, only the following variables were related to enhanced fibrinolysis (reduced delta LT): energy application (p = 0.039) and sinus rhythm at follow-up (p = 0.009). None of the other variables, including all clinical, procedural or haematological parameters listed in Tables 2 and 3, correlated with enhanced fibrinolysis. Multivariate analysis showed that sinus rhythm at follow-up remained a predictor of enhanced fibrinolysis after adjustment for other risk factors (p = 0.028).

### Effect of cardioversion on thrombotic status

**Fig. 4:**
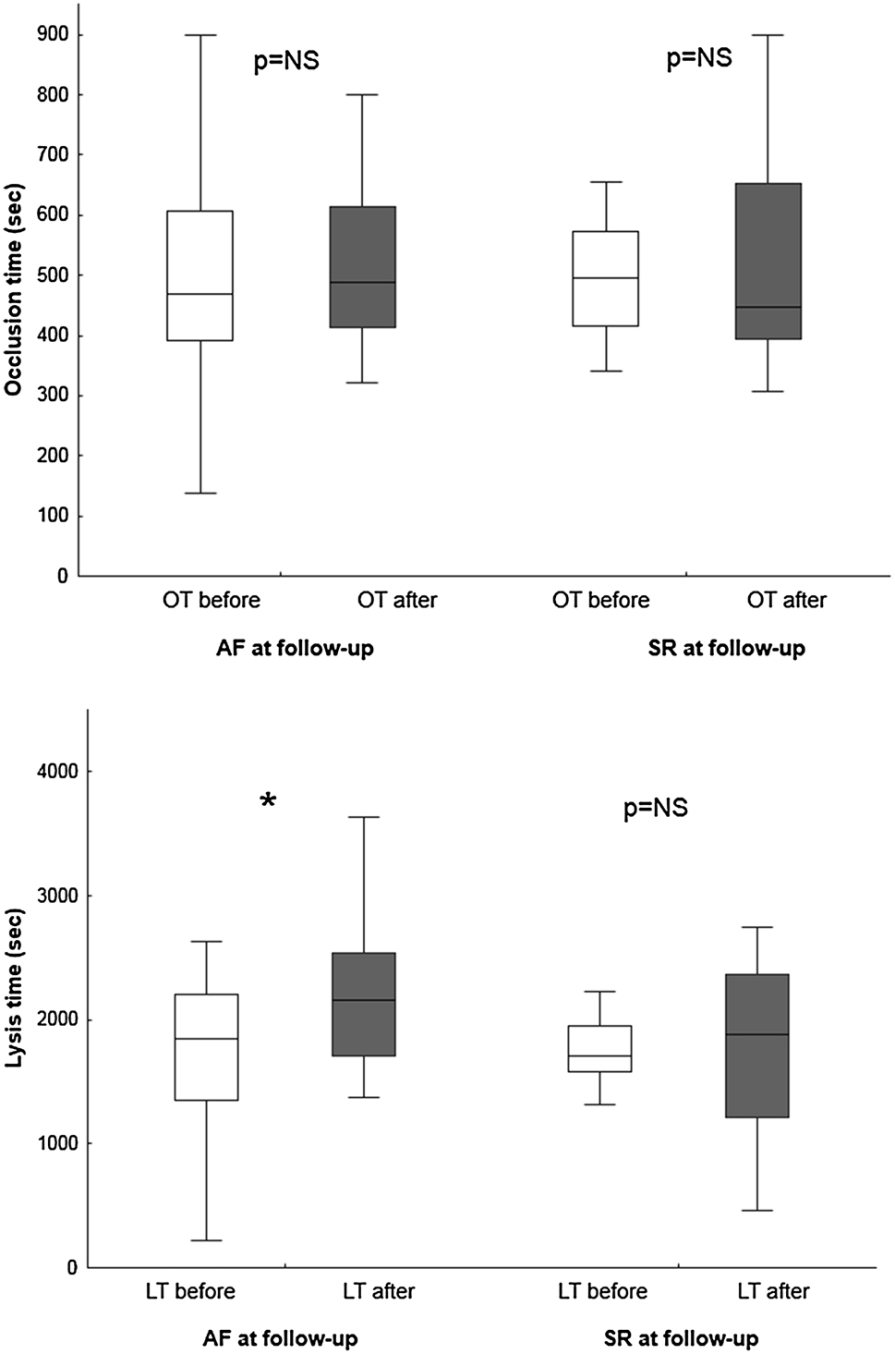
Occlusion time (OT) before and after cardioversion, grouped with respect to rhythm at follow-up. Lysis time (LT) before and after cardioversion, grouped with respect to rhythm at follow-up. *p = 0.005. Sinus rhythm (SR)

In the cardioversion cohort, 20 of the 40 patients had reverted to AF at follow-up. In the group as a whole, there was no difference in OT before and after DCCV (median [interquartile range], 487s [395; 573] vs. 482s [398; 639]; p = 0.247), nor in the subgroup who were in AF at follow-up (468s [392; 606] vs. 488s [414; 613]; p = 0.601), nor in those in SR (496s [416; 573] vs. 447s [394; 652]; p = 0.232) (Figs. 3, 4 and supplementary Fig. 5). At follow-up, there was no difference in OT between those patients who were in AF and those in SR (490s [394; 656] vs. 511s [398; 718]; p = 0.786).

There was no difference in LT before and after DCCV (1819s [1453; 2180] vs. 2026s [1647; 2367]; p = 0.052). However, compared to baseline, those who were in AF at follow-up demonstrated longer LT after DCCV (1852s [1346; 2208] vs. 2156s [1713; 2542]; p = 0.005), whereas there was no difference in LT before and after DCCV in those in SR at follow-up (1711s [1579; 1951] vs. 1877s [1216; 2367]; p = 1.000) (Fig. 4). At follow-up, there was no significant difference in LT between patients in AF and those in SR (2156s [1713; 2542] vs. 1877s [1216; 2367]; p = 0.14).

There was no difference between patients pre-and post-DCCV with respect to CRP (3.8 ± 4.53 vs. 4.46 ± 6.19; p = 0.467) or fibrinogen (4.1 ± 1.14 vs. 3.67 ± 1.18; p = 0.115). In the subgroup who reverted to AF, there was no difference in CRP (4.25 ± 4.71 vs. 3.85 ± 3.99; p = 0.776) or fibrinogen (3.93 ± 1.06 vs. 3.77 ± 1.38; p = 0.733) before and after the procedure; similarly, amongst those who maintained SR, there was no difference in either CRP (3.35 ± 4.42 vs. 5.1 ± 7.95; p = 0.158) or fibrinogen (4.28 ± 1.21 vs. 3.75 ± 1.08; p = 0.089) before and after the procedure.

In the DCCV group, using univariate linear regression analysis, only the following variables were related to enhanced fibrinolysis (reduced delta LT): high platelet count (p = 0.035) and angiotensin-converting enzyme inhibitor treatment (p = 0.002). None of the other variables including all clinical, procedural or haematological parameters in Tables 2 and 3 correlated with enhanced fibrinolysis.

### Central and peripheral assessment of thrombotic status

Patient and procedural characteristics are shown in Supplementary Table 1 and thrombotic status in Supplementary Table 2. Amongst patients with AF or left sided atrial tachycardia, there was no difference in OT or LT between blood samples from the femoral vein, right atrium or left atrium. Immediately after ablation, OT was significantly and profoundly prolonged, likely due to the effect of heparin, such that occlusive thrombus formation did not occur within the cut-off time of the test (800 s) and therefore LT also could not be measured (recorded as LT >6000s). Ablation was not reflected in an acute change in OT or LT at 4 h after ablation compared to baseline, with the exception of AF patients, in whom heparin was administered after the baseline sample and where 4 h later, OT was prolonged, likely an effect of heparin, rather than the ablation.

## Discussion

In this small exploratory study, successful treatment and restoration of SR in patients with AF using RFCA, is associated with improved global thrombotic status as evidenced by enhanced fibrinolysis. This improvement was not seen in patients who had restoration of SR using DCCV. Our study is hypothesis generating and suggests that altering the substrate of AF, through RFCA, not only improves the likelihood of maintaining SR, but may favourably alter thrombotic status.

A reduction of thrombotic risk would be expected with return of SR, in light of the restoration of effective mechanical atrial contraction and reduction in stasis. Prior studies have raised the possibility that AF ablation may reduce thrombotic risk [12, 13]. A registry comparing 4212 patients undergoing AF ablation to 16,848 matched controls with AF but without ablation, showed that AF patients treated with ablation had much lower risk of death, stroke, and dementia over 3-year follow-up, compared to AF patients without ablation [5, 8]. Furthermore, patients with AF ablation had similar long-term risk of stroke as patients without AF. This study however was not blinded or randomized, and the ablation arm had much more vigorous management than the control arm. A retrospective analysis of 24,244 patients with AF undergoing DCCV or RFA, showed that long term, ablation is associated with a lower rate of death, stroke or transient ischaemic attack than DCCV [12]. Two ongoing studies, the catheter ablation versus anti-arrhythmic drug therapy for atrial fibrillation trial (CABANA) trial (clinicaltrials.gov identifier NCT00911508), a prospective, randomized trial comparing RFCA with optimal medical therapy and EAST (early treatment of atrial fibrillation for stroke prevention trial, clinicaltrials.gov identifier NCT01288352) comparing “early” RFCA and antiarrhythmic medications vs. current standard practice, will help define the role of ablation in reducing adverse clinical outcomes.

The DCCV cohort was sampled at 6 weeks and the RFCA cohort at 3 months. 3 months is the conventional period to assess RFCA outcome; however, we arranged DCCV follow-up at 6 weeks because of concern that beyond this, few patients would still be in SR. Thus, sampling times are not directly comparable in the two groups and the lack of improvement in LT following DCCV could be attributed to “stunning”, since it may take many weeks of restoration of mechanical contractility, not just electrical restoration of SR, to observe changes in thrombotic status. One could also postulate that the RFCA group may have more complete freedom from *paroxysmal* atrial arrhythmias. The role of inflammation in the genesis and perpetuation of AF is increasingly recognized, with increasing AF burden associated with increasing CRP [3, 14]. High-sensitivity CRP decreases after successful RFCA for long-standing persistent AF, suggesting that AF itself may cause an inflammatory response [14, 15]. This may explain why RFCA but not DCCV improved thrombotic profile, although conflicts with the lack of change in CRP following successful RFCA in our cohort.

Our study showing impaired endogenous fibrinolysis in AF that is improved by restoration of SR, is supported by data on circulating D-dimer levels. The hypercoagulable state of AF is mirrored by increased levels of circulating D-dimer, a product of cross-linked fibrin degradation, reflecting activation of coagulation, fibrinolysis, or both. D-dimer levels are raised in AF and may identify those at high thromboembolic risk [3]. In small studies, both cardioversion and RFCA caused an early rise in D-dimer within 1–3 days, but the late effects of RFCA are less well documented. Lim showed that neither fibrinogen nor D-dimer levels fell 30 days after successful AF ablation, despite maintenance of SR, although a rise in fibrinogen level was associated with AF recurrence [16]. After successful AF ablation, some studies show CRP levels decrease significantly [15] whilst others show no change.

The lack of change in platelet reactivity (OT) in response to RFCA, conflicts with an earlier study where maintenance of SR 6 months after RFCA, was associated with reduced platelet activation and improvement in endothelial function [17]. However, whilst some studies have shown enhanced platelet activation in AF, others have not, or have documented only a local, and not systemic, phenomenon [2]. The role of platelet activation in AF therefore remains contentious.

Thrombotic status was similar in the left atrium and systemic circulation, and is at odds with some earlier work, showing that platelet reactivity was increased in the left atrium compared to the peripheral circulation [18, 19]. However, impaired endogenous thrombolysis in the systemic circulation is supported by a large volume of published data on the importance of circulating D-dimer levels in AF.

Limitations of our study include the fact that the DCCV cohort was assessed at 6 weeks and the RFCA cohort at 3 months. Thus, any differences in thrombotic status between those who maintained SR after DCCV and after RFCA, may simply be due to timing, perhaps due to incomplete restoration of left atrial mechanical contractility. We chose to sample patients post-RFA at 3 months because this is conventionally regarded as the end of the “blanking period”. During the first 3 months after AF ablation, the so-called “blanking period”, a substantial proportion of patients experience early recurrences of atrial tachyarrhythmias [20, 21]. Such early recurrences are thought to be due to transient local inflammatory states unassociated with the risk of later AF recurrence, and thus do not necessarily represent treatment failure [20]. It has, therefore, become common practice to consider a periprocedural blanking period, during which recurrences are considered nonspecific and do not prompt reintervention. The Heart Rhythm Society/European Heart Rhythm Association/European Cardiac Arrhythmia Society expert consensus recommends using 3 months as the conventional post-procedural blanking period, as recurrence within the first 3 months after ablation is not significantly associated with procedural success or failure as up to 65% of patients with AF recurrence in the first few months post-ablation will not have any further arrhythmias during long-term follow up [21]. However, the problem is that post-cardioversion, many patients revert to AF by 3 months, so we had to bring forward the sampling point to 6 weeks post-DCCV to capture as many patients post-DCCV in sinus rhythm, as possible. Our DCCV and cardioversion cohorts were generally well matched but there were some differences, such as NOAC use. Another possible confounder is the difference in anticoagulant treatment type between the DCCV and the RFA groups. However, our study was done at a time when AF ablation was not routinely performed on NOACs and thus the sample population reflected clinical practice. However, whilst such differences in OAC may affect between group comparisons (between DCCV and RFA), it should not affect within-group comparisons (namely thrombotic status before and after DCCV, or before and after RFA) since patients acted as self-controls and continued on the same OAC treatment throughout. Furthermore, we recently compared the relative effects of different non-vitamin K antagonist oral anticoagulants (rivaroxaban, dabigatran and apixaban) and warfarin, on global thrombotic status in patients with AF. We showed that NOACs and warfarin had a similar favourable effect on reducing platelet reactivity (evidenced by similar prolongation of OT with all agents). All NOACs exhibited a trend toward enhancing endogenous thrombolytic status (as evidenced by reduced LT), this was significant only for apixaban [22]. Therefore, although there are differences in the type of OAC used between the two cohorts, the impact of this on the results of thrombosis tests is likely to be non-significant.

Arrhythmia relapse reporting was based on symptoms and 24-h Holter monitor and/or follow-up ECG. It is thus possible that some patients experienced paroxysms of AF during follow-up but were misclassified as arrhythmia-free. Our patients were a mixed cohort of paroxysmal and persistent AF. Patients with paroxysmal AF may have intermediate levels of fibrin D-dimer compared to those with chronic AF or in SR which likely relates to fibrinolytic potential and thus the heterogeneous nature of our cohort may thus have been a potential confounder. However, against this is the finding that whilst the ratio of patients with paroxysmal AF was different between the DCCV and the RFA cohorts, OT and LT at baseline did not differ between the DCCV and RFA groups (Table 2). Furthermore, there was no difference in baseline OT or baseline LT between patients with different types of AF (paroxysmal, persistent, long-standing persistent). The GTT assesses platelet response to high shear, which does not replicate the low-flow state in the left atrium of patients with AF. However, unlike most low-flow states such as deep venous thrombosis where erythrocyte-rich thrombi prevail, in AF platelets contribute significantly to thrombus formation and the endothelial dysfunction which exists, suggests that an in vitro model like the GTT, where platelets and von Willebrand factor are key players and where endogenous fibrinolysis is assessed, may provide a useful assessment of this milieu. Furthermore, even if formed at low-flow states in the left atrium, such thrombi are subject to arterial flow, and our data and that of others suggest a systemic circulatory prothrombotic state and not just a localized left atrial phenomenon. The importance of endogenous fibrinolysis as a natural defence mechanism determining the outcome of thrombus formation is well recognized [23]. There are many physiological limitations to using single biomarkers to assess endogenous fibrinolysis, and emerging novel global assays of fibrinolysis such as used here have the potential to give a better overall assessment of thrombotic risk [23].

## Conclusion

Successful restoration and maintenance of SR following RFCA of AF is associated with improved global thrombotic status as evidenced by enhanced endogenous fibrinolysis. Our pilot data indicate the need for future large studies to investigate whether maintenance of SR with RFCA can reduce thrombotic risk in patients with AF.

## Electronic supplementary material

Below is the link to the electronic supplementary material.

Supplementary material 1 (DOCX 128 KB)
