# Supplementary material for: Catheter ablation for AF improves global thrombotic profile and enhances fibrinolysis
Source: J Thromb Thrombolysis. 2017 Sep 18;44(4):413–26. doi: 10.1007/s11239-017-1548-3 (PMC5658452; doi:10.1007/s11239-017-1548-3)

**Supplementary Table 1.**

Clinical characteristics of the study patients.

Values are mean ± standard deviation or n (%).

ACE: angiotensin-converting enzyme, ARB: angiotensin receptor blocker, BMI: body mass index, eGFR: estimated glomerular filtration rate; INR: international normalized ratio, LA: left atrium, N/A: not applicable , TIA: transient ischaemic attack

Normal values: haemoglobin 130-180 g/L in men and 115-165 g/L in women; haematocrit 40-52% in men and 36-47% in women; platelet count 150-400 x10^9^/L; INR range 2.0-3.0; creatinine 60-110 μmol/L in men and 45-90 μmol/L in women; eGFR: above 90mL/min/1.73m^2^; total cholesterol <5.2 mmol/L.

| **Patient**  **characteristics** | **Atrial fibrillation** | **Left-sided atrial**  **tachycardia** | **P value** |
| --- | --- | --- | --- |
| Number of patients | 15 | 5 |  |
| Age (years) | 68.4 ± 13 | 53 ±17 | 0.053 |
| Male gender | 9 (60) | 3 (60) | 1 |
| Body mass index (BMI) | 29.2±3 | 31.5±5.5 | 0.354 |
| Duration of arrhythmia >1 year | 10 (66) | 4 (80) | 0.319 |
| Duration of arrhythmia > 5 years | 6 (40) | 3 (60) | 0.342 |
| Previous ablations | 7 (46) | 4 (80) | 0.298 |
| Previous cardioversions | 3 (20) | 0 (0) | N/A |
| Co-morbidities |  |  |  |
| Coronary artery disease | 3 (20) | 1 (20) | 0.838 |
| Hypertension | 3 (20) | 1 (20) | 1 |
| Diabetes | 3 (20) | 0 (0) | N/A |
| Prior stroke/ TIA | 2 (13) | 0 (0) | N/A |
| Hyperlipidemia | 9 (60) | 3 (60) | 0.536 |
| Chronic kidney disease  (eGFR<60 mL/min/1.73 m^2^) | 1 (6) | 0 (0) | 0.577 |
| Medications |  |  |  |
| Beta-blockers | 9 (60) | 2 (40) | 0.098 |
| Calcium channel blockers | 3 (20) | 0 (0) | N/A |
| Flecainide | 1 (6) | 1 (20) | 0.565 |
| Amiodarone | 3 (20) | 2 (40) | 0.283 |
| Warfarin | 15 (100) | 3 (60) | 0.001 |
| Aspirin | 0 (0) | 2 (40) | N/A |
| Clopidogrel | 0 (0) | 0 (0) | N/A |
| Statins | 9 (60) | 3 (60) | 0.536 |
| ACE inhibitors | 1 (6) | 3 (60) | 0.092 |
| ARB | 3 (20) | 0 (0) | N/A |
| Echocardiographic parameters |  |  |  |
| Ejection fraction >55% | 12 (80) | 4 (80) | 0.595 |
| LA diameter < 4 cm | 5 (33) | 2 (40) | 0.955 |
| Laboratory parameters |  |  |  |
| Haemoglobin (g/dL) | 13±2 | 14±1.3 | 0.388 |
| Haematocrit (L/L) | 0.39±0.05 | 0.4±0.03 | 0.412 |
| Platelet count (x10^9^/L) | 197±42 | 218±64 | 0.406 |
| INR | 2.2±0.3 |  | N/A |
| Creatinine (μmol/L) | 87±24 | 89±28.7 | 0.901 |
| eGFR (mL/min/1.73 m^2^) | 83.4±23 | 101±46 | 0.249 |
| Total cholesterol (mmol/L) | 5±1.3 | 4.8±1 | 0.876 |

**Supplementary Table 2.**

Thrombotic status in intra-cardiac chambers and systemic circulation, of patients undergoing ablation for various arrhythmias. N/A: not applicable. Values represent Median (Interquartile range). *represents significant difference from baseline value. †800 sec was the cut-off time for OT. § 600 sec was the cut-off time for LT.

| **Occlusion Time** | **Baseline** | **End of ablation procedure** | **4 h post ablation** | **3 months post-ablation** |
| --- | --- | --- | --- | --- |
| **AF patients** | | | | |
| Peripheral vein  (ante-cubital fossa) | N/A | N/A | 557 (470; 756)  (p=0.049) * | 484 (420; 613)  (p=0.954) |
| Femoral vein | 490 (440; 549) | 800† (800; 800)  (p=0.001)* | N/A | N/A |
| Right atrium | 521 (406; 627) | 800 † (800; 800) (p=0.001)* | N/A | N/A |
| Left atrium | 585 (474; 658) | 800 † (800; 800)  (p=0.001)* | N/A | N/A |
| **Left sided atrial tachycardia** | | | | |
| Peripheral vein  (ante-cubital fossa) | N/A | N/A | 364 (308; 393)  (p=0.079) | 499 (427; 751)  (p=0.138) |
| Femoral vein | 485 (416; 489) | 800 † (800; 800)  (p=0.043)* | N/A | N/A |
| Right atrium | 489 (480; 558) | 800 † (800; 800)  (p=0.067) | N/A | N/A |
| Left atrium | 507 (479; 634) | 800† (800; 800)  (p=0.06) | N/A | N/A |

| **Lysis Time** | **Baseline** | **End of ablation procedure** | **4 h post ablation** | **3 months post-ablation** |
| --- | --- | --- | --- | --- |
| **AF patients** | | | | |
| Peripheral vein  (ante-cubital fossa) | N/A | N/A | 4461 (2188; 6000) (p=0.068) | 1921 (1060, 2328) (p=0.363) |
| Femoral vein | 2025 (1453; 2692) | 6000 § (6000; 6000) p=0.002)* | N/A | N/A |
| Right atrium | 2040 (1338; 5747) | 6000 § (6000; 6000) (p=0.054) | N/A | N/A |
| Left atrium | 2354 (1963; 3659) | 6000 § (6000; 6000) (p=0.004) * | N/A | N/A |
| **Left sided atrial tachycardia** | | | | |
| Peripheral vein  (ante-cubital fossa) | N/A | N/A | 1978 (685; 3659)  (p=0.5) | 1322 (1296; 1497) (p=0.079) |
| Femoral vein | 1790 (1790; 2056) | 6000 §(6000; 6000) (p=0.144) | N/A | N/A |
| Right atrium | 1685 (1676; 2577) | 6000 § (6000; 6000) (p=0.067) | N/A | N/A |
| Left atrium | 1652 (1589; 1722) | 6000 § (1376; 6000) (p=0.224) | N/A | N/A |

Supplementary Figure 5.

Distributions of occlusion time (OT) before (A) and after direct current cardioversion (DCCV) (B); distribution of lysis time (LT) before (C) and after DCCV (D). Y-axis shows number of observations (subjects).


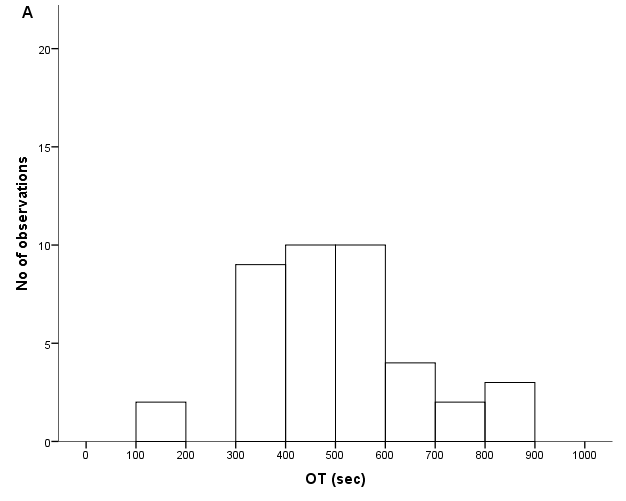

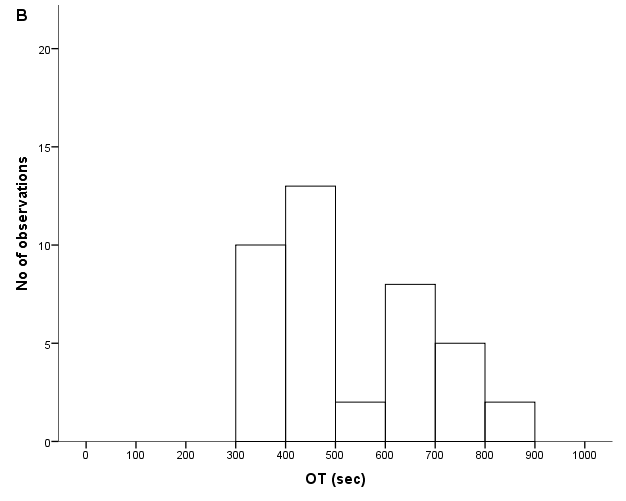

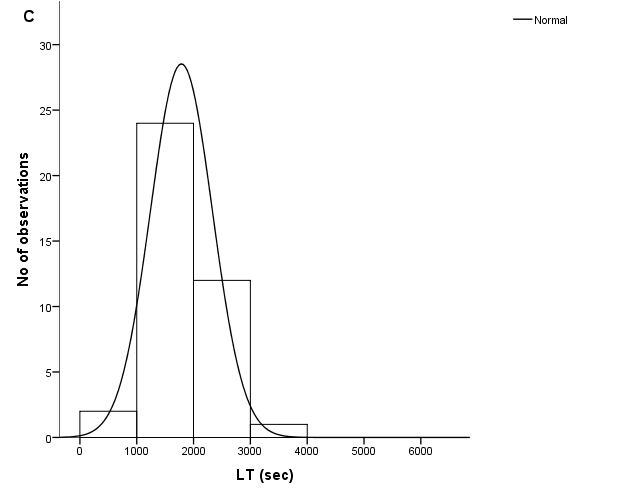

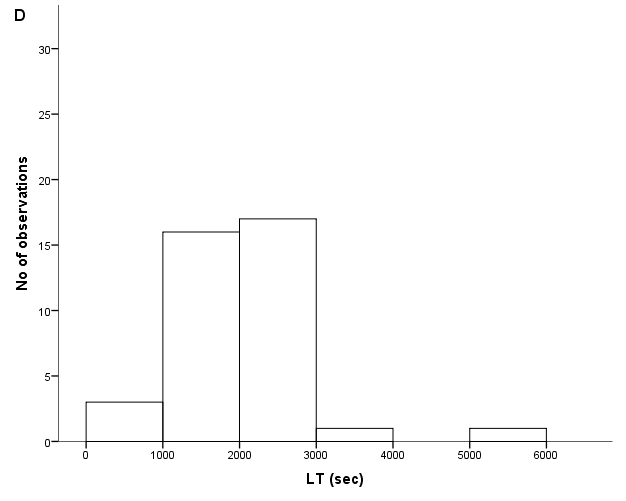

Supplement: Supplementary file 1 — Supplementary material 1 (DOCX 128 KB) [file 11239_2017_1548_MOESM1_ESM.docx]
